# Real-time detection and identification of nematode eggs genus and species through optical imaging

**DOI:** 10.1038/s41598-020-63747-5

**Published:** 2020-04-29

**Authors:** Farah Qazi, Asma Khalid, Arpita Poddar, Jean-Philippe Tetienne, Athavan Nadarajah, Arturo Aburto-Medina, Esmaeil Shahsavari, Ravi Shukla, Steven Prawer, Andrew S. Ball, Snjezana Tomljenovic-Hanic

**Affiliations:** 1 School of Physics, University of Melbourne, Parkville, 3010 Australia; 2 College of Science, Engineering and Health, RMIT University, Melbourne, Victoria, 3001 Australia; 3 Ian Potter NanoBiosensing Facility, NanoBiotechnology Research Laboratory (NBRL), School of Science, RMIT University, Melbourne, Victoria, 3001 Australia; 4 Centre for Environmental Sustainability and Remediation (EnSuRe), School of Science, RMIT University, Bundoora, Victoria, 3083 Australia

**Keywords:** Biological fluorescence, Single-molecule biophysics, Environmental sciences, Optics and photonics

## Abstract

Nematode eggs are pervasive pathogens that infect billions of people and livestock every year. Adult parasitic nematode worms can be distinguished based on their size and morphology. However, their eggs, particularly their species *Ascaris lumbricoides* and *Ascaris suum* cannot be identified from each other. Identifying eggs of helminths from wastewater and sludge is important from a public health perspective to minimize the spread of *Ascaris* infections. Numerous methods exist for nematode identification, from a morphological-based approach to high throughput sequencing technology. However, these techniques are not consistent and often laborious and time-consuming. In this study, we demonstrate that non-invasive real-time identification of eggs is possible based on their intrinsic fluorescence. Using confocal microscopy, we investigate the autofluorescence properties of five species of nematode eggs and observe clear differences between genus and for the first time their species in sludge samples. This non-invasive imaging technique could lead to better understanding of these species and may assist in early control of diseases.

## Introduction

The soil-transmitted helminths (parasitic worms) are causative agents of *helminthiasis* disease that infect 2.5 billion people across the globe^1^. This infection is predominantly present in resource-limited settings located in tropical and subtropical countries of the world^2^. In both developed and under-developed countries, parasitic nematodes’ eggs that infect grazing ruminants are a major economic threat^3,4^. Among these helminths, eggs of *Toxocara canis* are widely present in dogs and cats and they are causative agents of human *toxocariasis* which is a zoonotic infection^5^. Similarly, *Parascaris equorum* and *Oxyuris equi* eggs are responsible for equine helminth infection^6,7^. Furthermore, in humans, an annual disease burden of more than 1.5 billion is caused by chronic nematode infection *ascariasis* which is a parasitic nematode infection of human gastrointestinal tract caused by a giant roundworm, *Ascaris lumbricoides*^8^. The closely related parasite *Ascaris suum* is a large roundworm of pigs and prevalence of infection varies depending on farm management practices^9^. Identification of parasitic helminth eggs is crucial for preventing and managing parasite diseases^1^. The widespread use of recycled water from wastewater treatment for irrigation and sludge reuse in agriculture systems potentially provides a means of spreading infection rapidly through a population. Parasitic helminths of the genera *Ascaris*, *Trichuris*, *Ancylostoma* and *Toxocara* are highly resistant to wastewater treatment processes^10^. Furthermore, *Ascaris lumbricoides* (human roundworm) and *Ascaris suum* (pig roundworm) ova exhibit increased resistance to adverse conditions, such as high temperatures and alkaline pH compared with other helminths^11^. Therefore, *Ascaris* eggs are used as an indicator of helminth egg presence since they are equally or more persistent than other genera eggs including *Trichuris trichiuria* and hookworms^11^. The identification and enumeration of *Ascaris lumbricoides* represents a key step in preventing the spread of infection. Being able to rapidly distinguish between human pathogen *Ascaris lumbricoides* and animal pathogens such as *Ascaris suum* will inform the public about health risks associated with the eggs and aid in tracking the source of contamination. The importance of quality-assured diagnostic tests for *helminthiasis* disease is often neglected and emphasis is always given on drug treatment and vaccination^12^. More sensitive and reliable identification methods of helminth eggs would lead to proper clinical diagnostic approach and further progress will be made towards ultimate control and elimination of these infections^12^. The difficulty in identification of these parasites is challenging due to only subtle differences in the morphology of eggs^9,13^. This requires sensitive method for detection and identification of helminth eggs for their complete control at species level^14^.

Over the last few years, various methods for detection and quantification of parasitic nematode eggs have been developed and applied but they are time-consuming and laborious^15,16^. One of these methods is polymerase chain reaction (PCR) in which DNA is extracted to carry out gene amplification; however, the flexible exoskeleton of pathogens is sensitive to mild physical or chemical disruption, hence DNA extraction is laborious and time-consuming^17^. Similarly, routine analytical procedures are commonly based on two steps for nematode identification. In the first the eggs are separated from other particles in wastewater. Then a trained technician visually identifies the different species of helminth eggs in their different life stages^18^.These methods are tedious, impractical and time-consuming and reliant on the expertise of the technician examining the eggs.

There have also been reports on the use of confocal imaging of nematode eggs for identification^19,20^. In this case live *Ascaris* and *Trichuris* eggs stained by LIVE/DEAD kit glow green and *Toxocara* eggs green-blue. On the other hand, all inactivated eggs are colored red. The studies verified that after few modifications the LIVE/DEAD kit is also helpful for viability assessment of *Toxocara, Ascaris* and *Trichuris* eggs found in the sludge. In another study, Karkashan *et al*. evaluated the LIVE/DEAD BacLight methodology for numbering viable eggs of *Ascaris suum* in sludge, which is assumed to be an effective substitute for the human round worms^19^. The BacLight method was effective for accurate examination of *Ascaris suum* eggs directly in sewage sludge^19^. However, the authors concluded that a recovery process would be essential to examine samples containing only small proportion of eggs.

In fact, environmental and biomedical scientists commonly use commercial confocal microscopy techniques that use dyes and proteins for tagging^21^. The fluorescent agents used in commercial confocal microscopy modify the chemistry of the analyte which may induce changes in the properties being examined such as eggs’ identification, development and transport^22^. Dyes also suffer from poor hydrophilicity and photostability^22,23^. Additionally, they exhibit low quantum yields with low detection sensitivity and have insufficient stability in biological systems^24^. In order to improve image efficiency, these dyes have been conjugated to other nanoparticles and biological macromolecules through chemical conjugation methods that in turn may compromise the imaging properties^22^. To date, only a few studies have been undertaken on the use of autofluorescence properties for microbial identification^25^. Some studies report bacterial and yeast characterization using intrinsic fluorescence^26^, but to the best of our knowledge, there has been no report on identification of nematode eggs based on their intrinsic fluorescence. Here we report a non-invasive method for identification of genus and for the first-time, species of nematode eggs i.e *Ascaris lumbricoides* and *Ascaris suum*, based on their specific fluorescence properties, namely emission spectrum and fluorescence lifetime.

We first investigated the optical and emission properties of the parasitic eggs using confocal microscopy at room temperature, then studied their fluorescence properties at cryogenic temperature (80 K) using Raman microscopy. We find that these eggs are non-invasively detectable by their intrinsic fluorescence and can be traced without the need for additional tags and dyes. We also identified them in sludge samples based on their optical properties. Our findings establish fluorescence microscopy as a promising method for identification of helminths in a real-time, non-invasive way without any requirement of fluorescent tags and dyes.

## Results and Discussion

The manuscript reports the identification of different species of nematode eggs using their intrinsic fluorescence. This work investigates and compares the fluorescence signatures of genus and species of nematode eggs. In section “Detection of nematode eggs”, we investigate the detection and tracking of nematode eggs using wide-field and confocal microscopy. Section “Identification of nematode eggs with photoluminescence spectral measurements” presents the methodology for the identification of nematode eggs using spectral measurements. Section “Further identification of nematode eggs-Lifetime measurements” provides an additional tool for the identification of genus and species of nematodes using lifetime measurements.

### Detection of nematode eggs

#### The nematode eggs imaging using wide-filed and confocal microscopy

##### ***Wide-field microscopy.***

**Figure 1:**
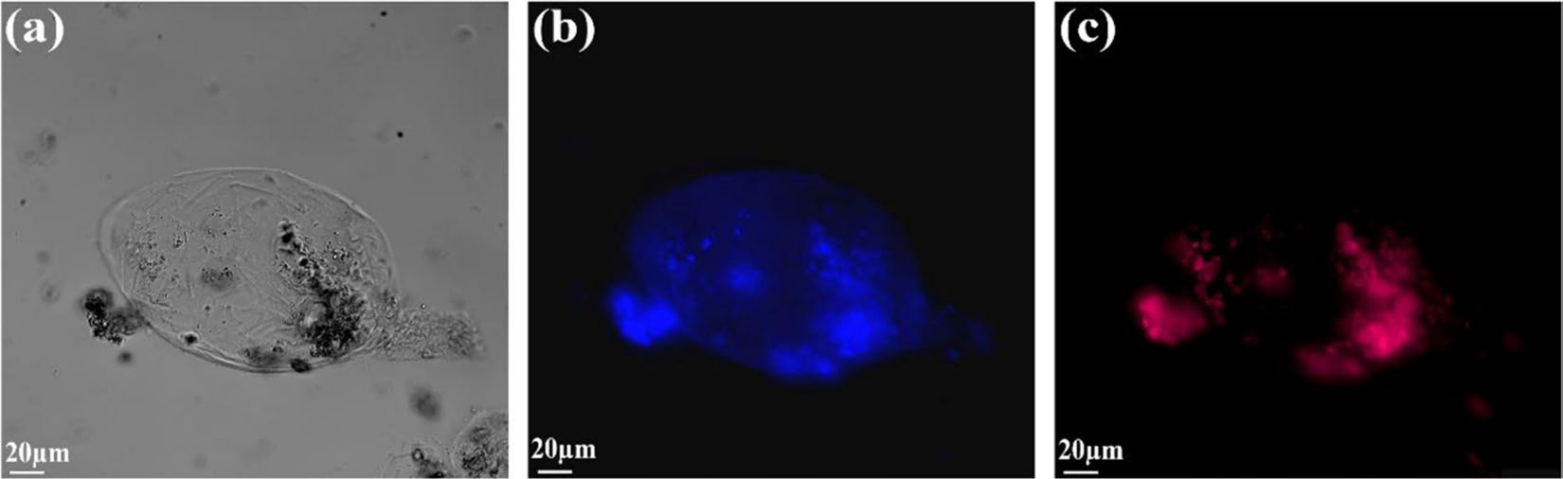
The wide-field confocal image of *Ascaris lumbricoides* egg under (**a**) white-light (**b**) 390 nm UV (**c**) 560 nm light illumination.

For the optical characterisation of nematode eggs, we used wide-field fluorescence microscopy. This experiment uses the intrinsic fluorescence from nematode eggs for their detection without the use of tags and dyes. Fig. 1 shows wide field images of nematode eggs under white light (a), UV (b), and green laser (c) excitation.

As shown in Fig. 1(a), the egg of *Ascaris lumbricoides* can easily be visualized through light microscopy. Moreover, they are fluorescent in the visible region when illuminated with 390 nm (Fig. 1(b)), and 560 nm laser light (Fig. 1(c)). The eggs show fluorescence due to presence of lipids and proteins in their cell-membrane as discussed in section “Identification of species”. We also perform wide-field microscopy experiment on other species of nematode eggs, that indicate they are fluorescent across wide range of spectrum (see Supplementary Fig. 1–4). However, light microscopy alone doesn’t provide enough information to enable the identification of nematode genus and species. So, other techniques have been used to detect and to identify nematode eggs and juveniles as discussed in section “Identification of nematode eggs with photoluminescence spectral measurements” and “Further identification of nematode eggs-Lifetime measurements”.

##### **Confocal microscope.**

Confocal microscope has an in plane resolution of 300 nm, but even smaller fluorescent features are detectable. The size of nematode egg ranged from 40 µm to 150 µm tha can easily be detected using confocal microscope. The optical analysis of eggs was performed using a home-built confocal microscope (see Section “Optical characterization”). Fluorescence scans of 100 µm × 100 µm were taken, emission counts were recorded, and photostability were investigated.

**Figure 2:**
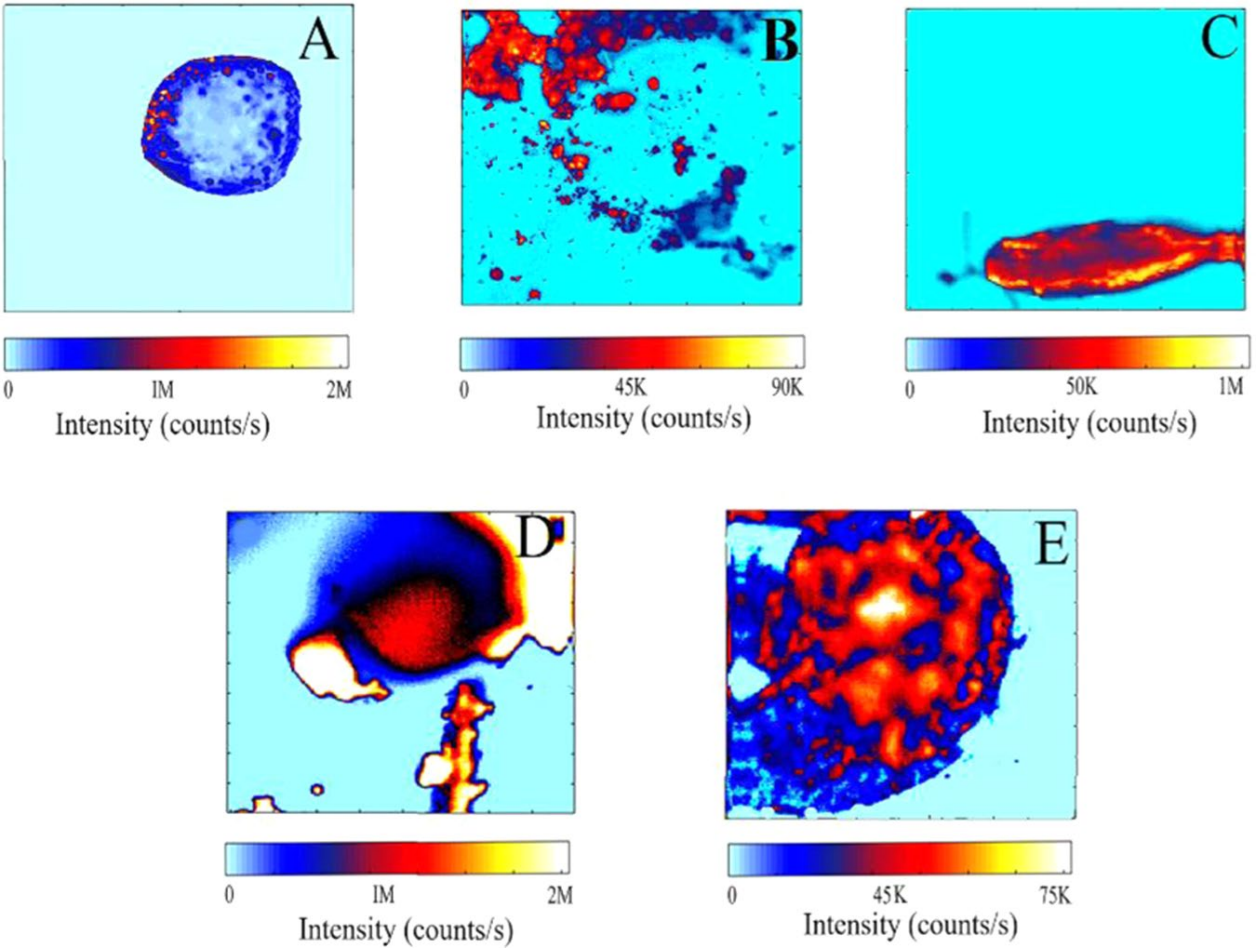
A 100 µm × 100 µm confocal fluorescence map for (**A**) *Ascaris lumbricoides* (**B**) *Ascaris suum* (**C**) *Oxyuris equi* (**D**) *Toxocara canis* and (**E**) *Parascaris equorum* taken at the power ranging from 20 µW to 50 µW. For the illumination of samples, 532 nm laser was used, and the fluorescence was filtered by a 532 nm notch filter before detection.

Representative confocal scans of nematodes are shown in Fig. 2 in which (a) represents eggs of *Ascaris lumbricoides*, recognized by their optically distinct rounded shape. The diameter of eggs was 40 μm with the feature size of emitters in the range of 4 µm to 6 µm respectively. These eggs showed more than 2.0 million (M) counts per second using a power of 25 µW, indicating the presence of very bright emitters in their structure. The power used is far below the (threshold) risk of photothermal damage to biological samples, yet strong emission from nematode eggs is observed. For comparison, non-treated nano-diamonds of similar size produce less than 10 times the brightness. Figure 2(B) shows representative autofluorescence confocal scan for *Ascaris suum* eggs. These eggs are bright with counts in the range of 0.09 M per second at power of 25 µW. These eggs have relatively large feature sizes of the order of 5 µm to 15 µm compared to *Ascaris lumbricoides* eggs. Figure 2(C) demonstrates characteristic bright fluorophores of *Oxyuris equi* eggs. The counts were recorded more than 1.0 M per second from each egg at a power of 20 μW. These eggs have oval shape with diameter of 80 μm. Figure 2(D) shows confocal scan for *Toxocara canis* also taken at power of 20 μW. Counts of 2 M per second were recorded and feature size of emitters is in the range of 50 µm to 60 µm. Figure 2(E) represents fluorescence image of *Parascaris equorum*. The counts observed for *Parascaris equorum* eggs were around 0.075 M per second with 20 µW power used. Morphologically they are also rounded in shape with significantly larger eggs size around 120 µm and fluorescent features of the order of 10 µm to 30 µm in their structure.

In these confocal scans, red fluorescence is observed in all these eggs structures, indicative of extremely bright fluorescent features even at the low laser power. These images show a high density of spatial and spectrally distinct fluorescence features originating from fluorophores present in the cell membrane of eggs. This bright red fluorescence is attributed to the presence of lipids and proteins layers in the cell membrane of eggs as discussed in section “Identification of species”^27–29^.

#### Juvenile imaging

**Figure 3:**
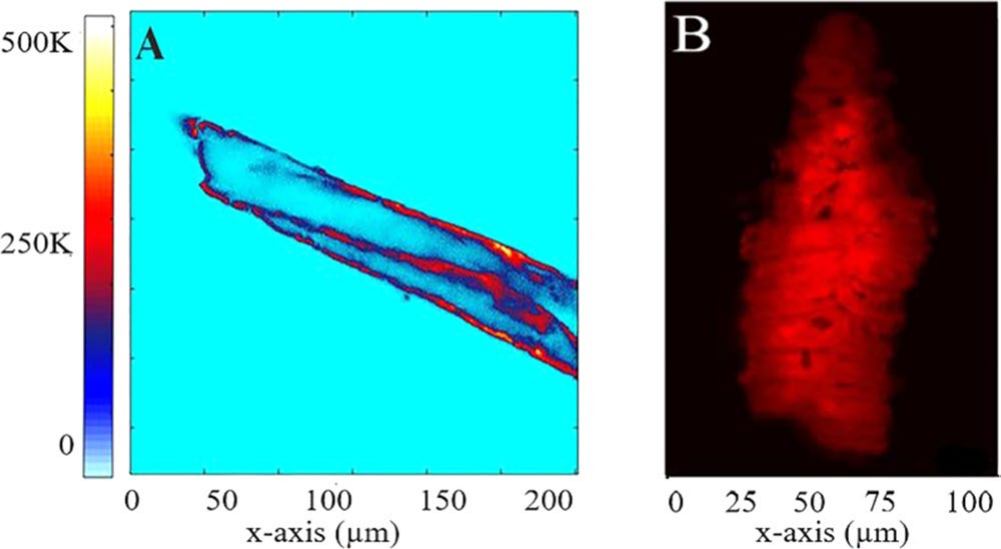
(**A**) Confocal map of *Ascaris suum* juvenile, showing red fluorescence from outer membranes after 532 nm laser excitation (**B**) Confocal image of *Ascaris lumbricoides* juvenile, showing red fluorescence after excitation with 561 nm.

Like eggs, juveniles of these pathogens are still difficult to differentiate precisely due to similar morphological features. To date, there have been a few studies for identification of parasitic nematode juveniles^30^. Those usually involve measurement of some of the conventional characteristics such as length of sheath tails of different species of juvenile^31,32^. In order to investigate fluorescence properties of nematodes juveniles, we have performed a confocal experiment on *Ascaris suum* and *Ascaris lumbricoides* juveniles as shown in Fig. 3.

**Figure 4:**
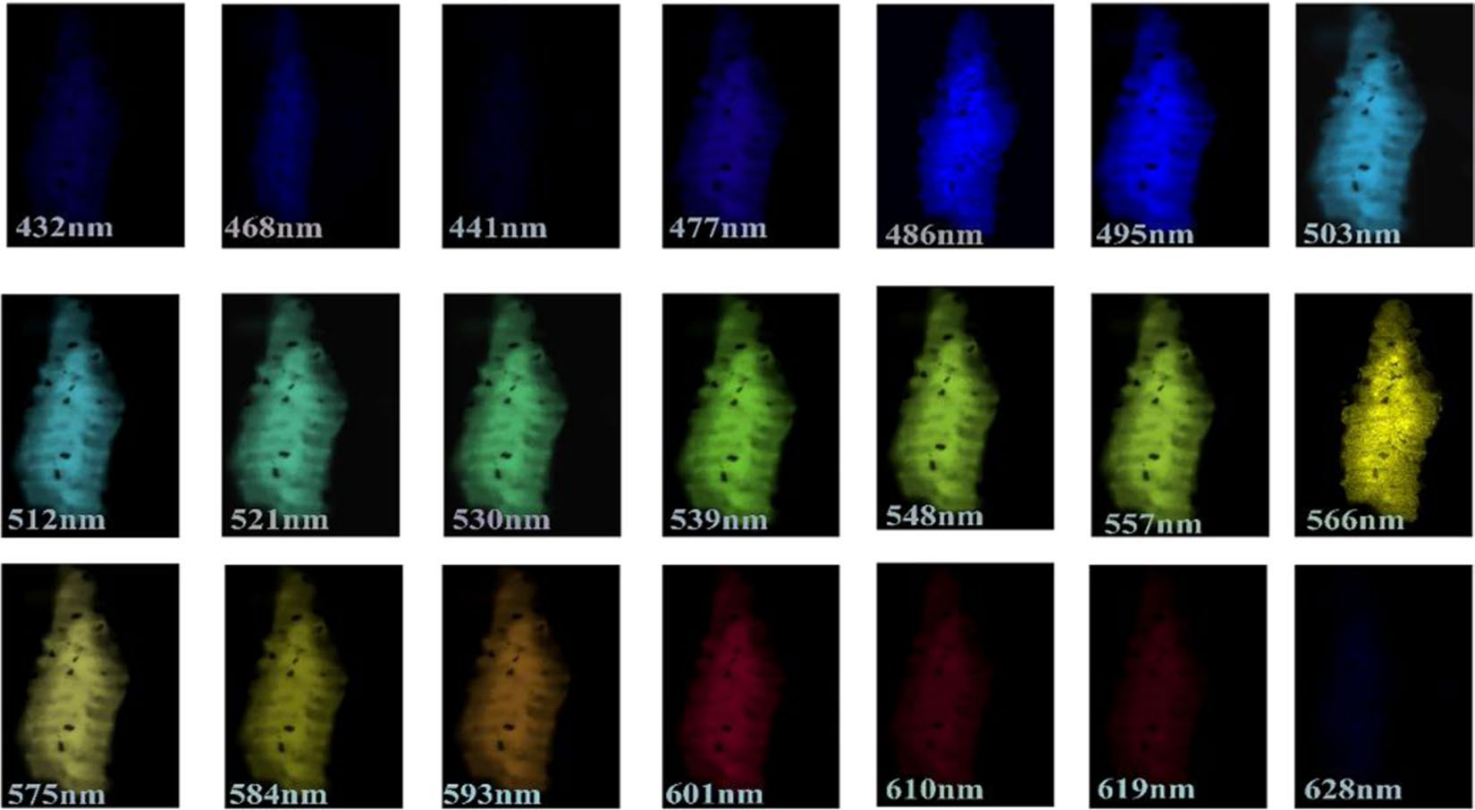
The observation of *Ascaris lumbricoides* larva after exposure to 405 nm wavelength in lambda mode of Zeiss confocal LSM 880. The fluorescence was observed in the range of 432 nm to 628 nm respectively.

**Figure 5:**
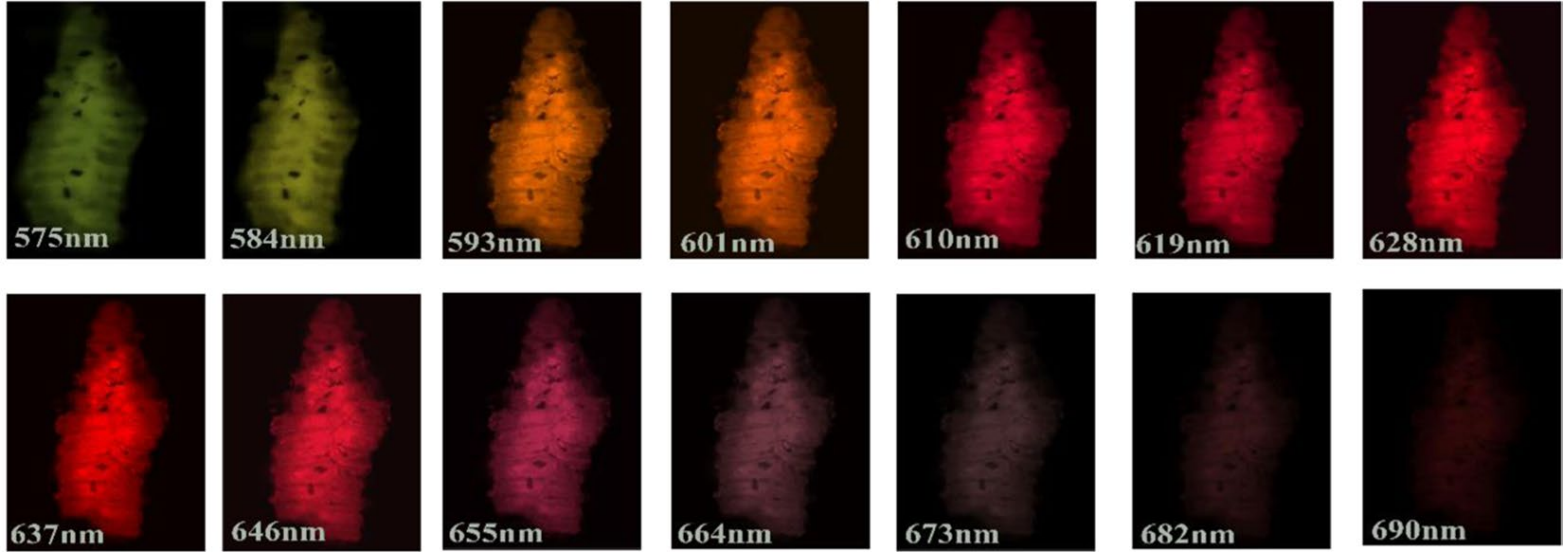
Fluorescence scan of *Ascaris lumbricoides* juvenile at 561 nm laser excitation, the red fluorescence was observed in lambda mode from 575 nm to 690 nm.

Figure 3(A) shows *Ascaris suum* juvenile elongated in shape, which have a distinct and spectrally resolved fluorescent membrane while Fig. 3(B) shows the 2*D* Zeiss confocal scan for *Ascaris lumbricoides* juvenile. Previous studies show that during the hatching of nematodes their collagen and lipids layers start to breakdown in its external structure, known as a cuticle, which is essential for their survival and development^33^. This cuticle is primarily composed of collagen, lipids and proteins with a small amount of carbohydrates^34^. The fluorescence properties in red, due to presence of these proteins and lipids in *Ascaris* juveniles as shown in section “Indentification of species”^34^. Fluorescent properties in juveniles persist with the same properties as observed for the eggs. Although these images are taken from a different setup, both juveniles showed auto-fluorescence in red when excited with wavelength of 532 nm and 561 nm respectively. However, their intrinsic fluorescence exists for a large range of wavelengths, as shown in Figs. 4, 5. Depending on the excitation wavelengths the maximum emission can appear at different wavelengths and colors other than red.

In conventional confocal methods, specific filter sets and linear unmixing steps are required to separate highly overlapping fluorescent labels and tags^35^. Hence, correct quantitative analysis of dataset become time consuming and labor intensive. To avoid this, we relied on the auto fluorescent properties of juveniles and obtained spectra of juveniles using two different setups.

In addition, z-stack was taken using Zeiss confocal microscope to investigate the fluorescence properties of *Ascaris lumbricoides* juveniles as shown in Fig. 4. The analysis of the surface auto fluorescent light (lambda mode) of the juvenile shows a broad range of emission between the boundaries of 432 nm and 628 nm after excitation with 405 nm laser respectively. The results show that *Ascaris lumbricoides* juvenile demonstrate very strong autofluorescence emission in the green region of the spectrum, around 503 nm to 566 nm; however their fluorescence is reduced in the blue and red regions which support our results from absorption spectra described in section “Identification of nematode eggs with photoluminescence spectral measurements”.

In contrast, exposure of sample to 561 nm wavelength shows that auto-fluorescence is dominant in the red region from 601 nm to 655 nm which is in support of emission spectra (Section “Identification of nematode eggs with photoluminescence spectral measurements”) results obtained using the confocal microscope as shown in Fig. 5. These results illustrate that choice of the excitation wavelength (laser) is extremely important for demonstration of the fluorescence characteristics of juveniles. Unlike conventional methods, linear mixing is not required as our experiment is based on the intrinsic fluorescence properties of juveniles. This method would enable generation of images and spectral details of complex biological samples without the use of fluorescence labeling, avoiding the use of subsequent linear unmixing steps and the complexity related to highly overlapping fluorophores into pure signals.

#### Tracking Ability-Photostability

Currently, there is not enough insight into the response of nematode and other parasitic eggs to environmental changes and drugs in real-time.

**Figure 6:**
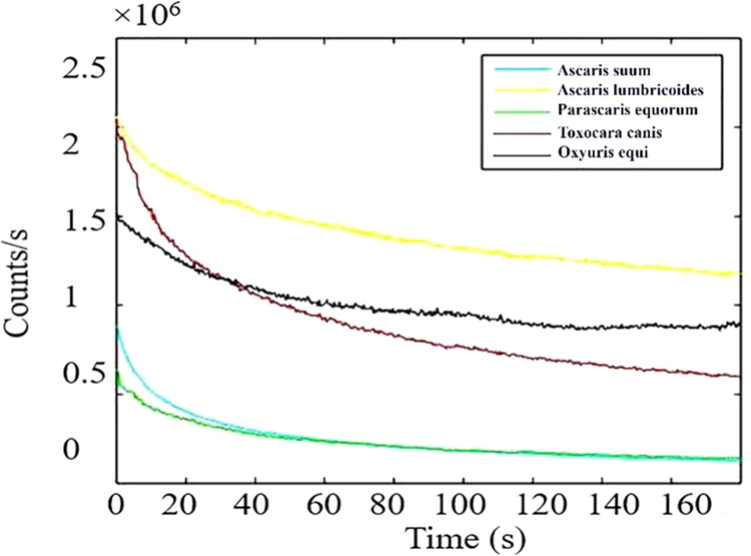
Photostability investigation: Representative fluorescence emission rates as a function of time for five parasitic nematode egg species. The fluorescence initially bleached for up to 20 s and then tends to become stable for 180 s without photobleaching at the power of 20 µW. The background (glass slide) counts were of the order of 2k per second.

In addition to morphological autofluorescence imaging, we also analyzed the time traces of the photoluminescence as a function of time for each egg sample as shown in Fig. 6. The photostability of fluorescing features is an important characteristic requirement for long-term bioimaging^36^. The stable count rate may be partly due to the lower power used for excitation. It is apparent from Fig. 6 that counts decrease initially, within a few seconds, before getting stable over the measured period (180 s). The initial photobleaching (20 s) might be attributed to some impurities attached to the surface of eggs. A similar trend of *in vitro* fluorescence was seen inside the human keratinocyte cells with the ZnO nanoparticles^37^. Figure 6 shows that *Ascaris lumbricoides* have the brightest fluorophores on its outer surface showing emission counts of more than 2 M counts per second. Similarly, initial counts observed in *Toxocara canis* was 2 M which became stable around 1 M after 20 s of photobleaching. Furthermore, *Oxyuris equi* show counts around 1.8 M per second. However, the counts for *Ascaris suum* and *Parascaris equorum* were recorded in the range of 0.09 M per second to 0.07 M per second respectively. The background fluorescence (light blue) is significantly less, of the order of 0.02 M counts per second, assisting clear identification of eggs. It is highly advantageous if the intrinsic fluorescence of these eggs can be used for tracking and recording without any additional tags. In this way, there are two essential advantages. One is that there is no induced change in the chemistry of these biosamples. Second, their natural kinematics and dynamics will not be affected with tags.

### Identification of nematode eggs with photoluminescence spectral measurements

**Figure 7:**
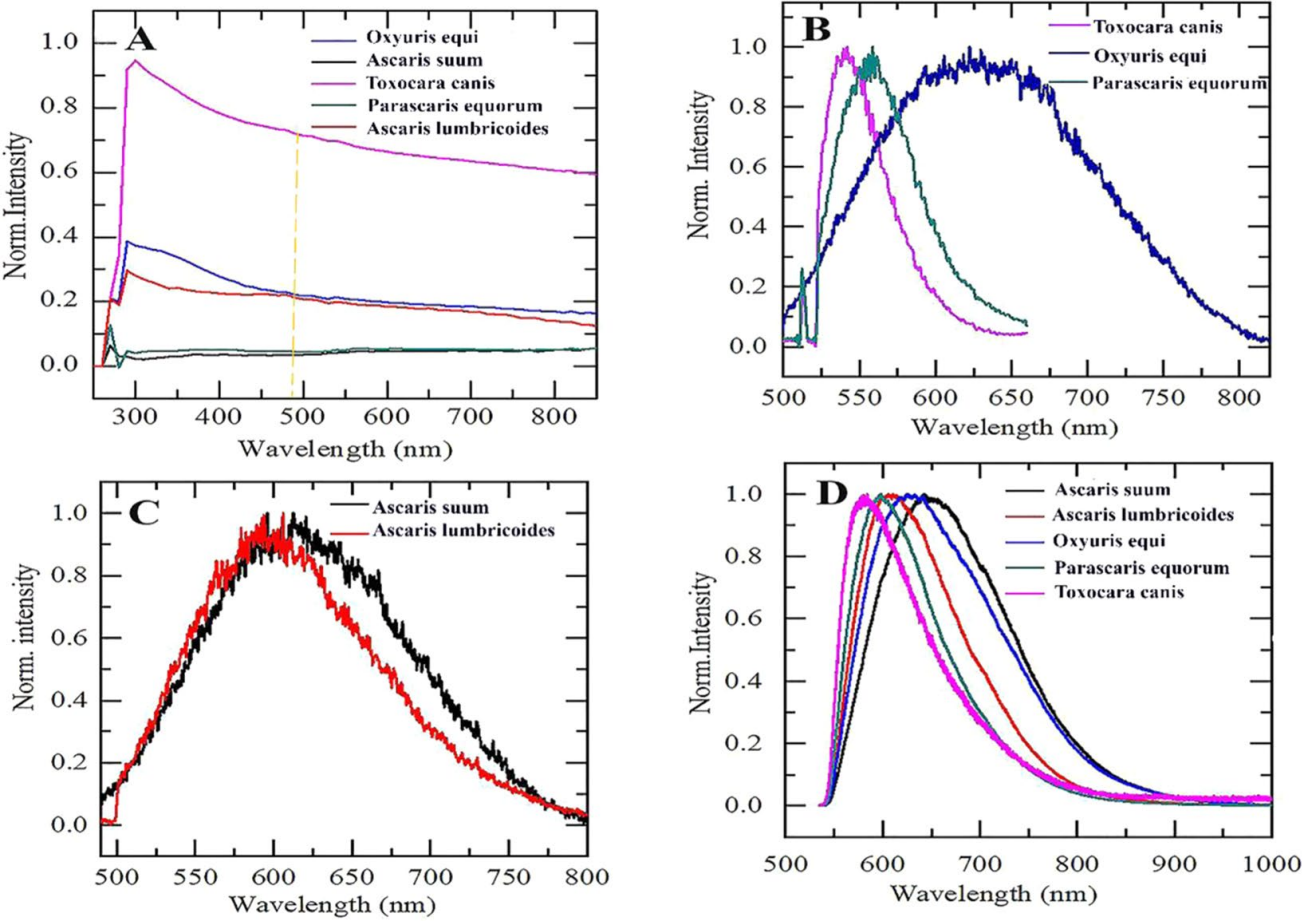
**(A)** Absorption spectra of different genus and species of nematodes: *Oxyuris equi* (blue), *Parascaris equorum* (green)*,Toxocara canis* (pink)*, Ascaris lumbricoides* (red) and *Ascaris suum* (black). The orange dotted line indicates the wavelength used for excitation of sample **(B)** Fluorescence emission spectra of different genus of nematodes. All spectra were acquired under illumination with a blue laser (480 nm) at the power range of 5 µW to 10 μW through 500 nm long pass filter using confocal microscopy. **(D)** Emission spectra of species i.e. *Ascaris lumbricoides* and *Ascaris suum* **(C)** Fluorescence emission spectra of different species of nematode eggs acquired by Raman microscopy using a 532 nm Argon laser at a temperature of 80 K.

Nematode eggs can be differentiated by optical imaging, not only in confocal but in other microscopic methods^38^. However, identification based on images is insufficient and time- and labour- consuming^16^. Selectivity can be maximized by detecting specific optical signatures in spectra of single species. In order to investigate the spectral properties of nematode eggs, their absorption spectra were recorded using Plate reader-SpectraMax Paradigm Molecular device, and emission spectra were recorded using confocal microscopy at room temperature and Raman microscopy at cryogenic temperature (see Section “Optical characterization”). As shown in Fig. 7(A) the recorded absorbance for nematode eggs increased at shorter wavelengths. The absorbance for all the species was in the visible to infrared range. These results indicate that there is a large range of wavelengths where an optical pump can be used to excite eggs fluorescent features.

The emission fluorescence spectra of eggs were measured from 480 nm to 800 nm, as presented in Fig. 7B. Spectral analysis of eggs was performed to determine the characteristic emission signature of fluorophores. *Oxyuris equi* eggs exhibit broad fluorescence from 500 nm to 800 nm (Fig. 7B). Furthermore, the characteristic emission peak for *Oxyuris equi* was observed at 630 nm. *Parascaris equorum* and *Toxocara canis* reveal characteristic features peaks at ~540 nm and 560 nm, respectively. Spectra for *Ascaris lumbricoides* and *Ascaris suum* were also taken under the same conditions, showing characteristic peaks at ~595 nm and 613 nm, respectively as shown in Fig. 7(B). Spectral analysis show that fluorophores present in these eggs are spectrally different due to the presence of different compounds in their structures as shown in section “Identification of species”.

All of the emission spectra measured at room temperature occur over a broad wavelength range. The results are presented in Fig. 7(D). For fluorescence analysis, nematode eggs were placed in a nitrogen-flow cryostat. The fluorescence spectra were collected with a microscope system with a 532 nm argon laser. The excitation laser was focused on the substrate and fluorescence spectra were acquired. With the intention to sharpen the characteristic peaks of parasitic eggs, we analyzed the effect of low temperature (80 K) on fluorescence emission spectra of nematodes. With decreasing temperature, the spectra remain unchanged, but a blue shift of 20 nm towards the lower temperature was recorded compared to room temperature. At this stage, we cannot assign a reason for the 20 nm blue shift in the fluorescence spectra of eggs under cryogenic conditions (Fig. 7D). Further investigation using a liquid helium temperature (~270 K) may be useful to see if a substantial change in photoluminescence properties of nematode eggs exist. Therefore, the expected sharpening at lower temperatures did not occur.

In this study, spectral results reveal that each species of nematode egg has distinct and resolvable emission spectra and these eggs can be distinguished through their spectroscopic fingerprint using a confocal at room temperature.

As mentioned in the introduction, only a few studies take advantage of the autofluorescence properties of bacteria in which their spontaneous emission spectra were recorded upon excitation at a specific wavelength^39^. The spectra measurement results shown in section “Identification of nematode eggs with photoluminescence spectral measurements” demonstrated that each species of nematode has a specific emission spectrum and can easily be distinguished from each other without fluorescent labels.

### Further Identification of nematode eggs - Lifetime Measurements

In addition to spectral features, lifetime measurement is the intrinsic property of an emitter and possible candidate for a single parameter-driven method of identification. For lifetime imaging, the decay in fluorescence intensity is recorded across the sample after optical excitation with a short laser pulse (6 ps) at 532 nm.

**Figure 8:**
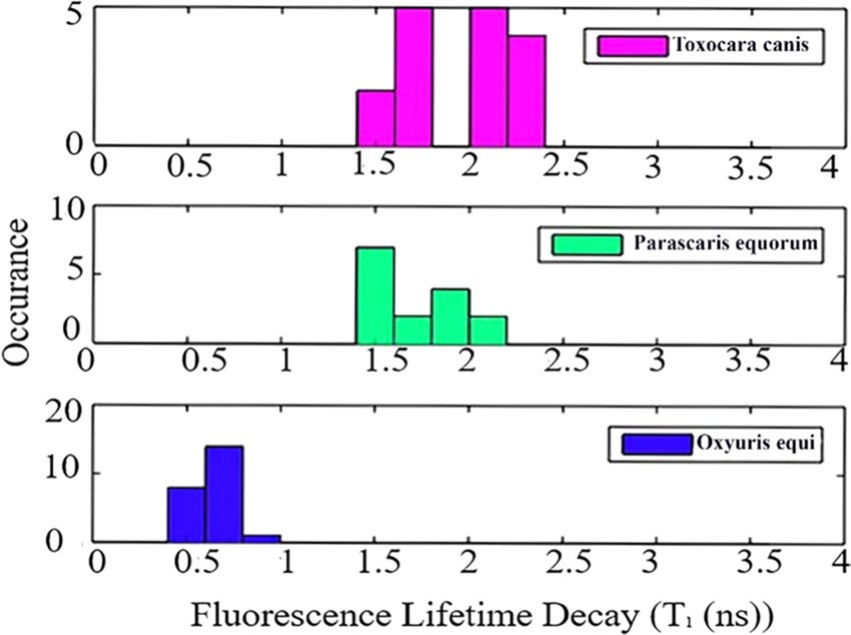
Histogram plot showing lifetime distribution, the data sets contain 15 to 23 different fluorescent sites from a total of three to four eggs of each sample. The figure shows lifetimes of *Toxocara canis* (pink), *Parascaris equorum* (green) and *Oxyuris equi* (blue). Samples were excited with 532 nm pulsed laser source, with the repetition rate of 20 MHz at an excitation power of 9 to 15 µW.

Figure 8 shows a comparison of histograms of lifetimes obtained from stretched exponential fit for different species. Each data set contained 15 to 23 fluorescent sites from a total of three to four different eggs. The mean fluorescence lifetime values of *Toxocara canis* and *Parascaris equorum* were approximately 1.9 ± 0.3 ns and 1.7 ± 0.2 ns (mean ± standard deviation), respectively. The data set contained 15 and 16 fluorescent sites from a total of three different eggs of *Parascaris equorum* and *Toxocara canis* respectively. In addition, the lifetime of *Oxyuris equi* was measured to be 0.6 ± 0.1 ns. It is likely that lipoproteins present in the eggshell has similar fluorophores and therefore lifetime is overlapping at certain points for some eggs. Further investigation is needed to fully understand the significance of these results. However, the significant difference observed between lifetime of *Toxocara, Parascaris and Oxyuris* in Fig. 8, suggested lifetime is a promising parameter for identification of different species of nematodes. Along with fluorescence spectra results (Section “Identification of nematode eggs with photoluminescence spectral measurements”), lifetime measurements of eggs can give more confidence for identification of unknown egg sample, leading to the development of effective diagnostic methods of helminths.

The nematode eggs of different species and genus, *Ascaris suum*, *Toxocara canis*, *Parascaris equorum*, were mixed, and analysed. We have differentiated different genus and species by obtaining their spectra and lifetime. The results remained consistent, confirming that, the different species of nematodes can be differentiated with high certainty. This indicates that this technique is highly applicable in formulation of a device which may be used for identification of nematode genus and species.

#### Identification of species

The two socioeconomic parasitic nematodes are *Ascaris lumbricoides* and *Ascaris suum*, infecting humans and pigs respectively, and are responsible for worldwide disease *ascariasis*^40^. The dispute on their taxonomical status and zoonotic origins has persisted for decades^41^. Beside taxonomy, another important epidemiological concern with the implementation of a control program of *ascariasis* disease, especially in areas where *Ascaris* coexist, is whether control programs should be applied for only infected humans, or for both infected animals and humans^42^. To address this issue, several different approaches have recently been used based on morphological^43^, biochemical^44^, karyotypic^45^, immunological^44^ and molecular methods^46^, but with limited success.

We performed Raman vibrational spectroscopy to collect biochemical information of the *Ascaris suum* and *Ascaris lumbricoides* eggs. The shell of *Ascaris lumbricoides* eggs have been previously investigated using Raman spectroscopy^47^; however, comparative spectra to differentiate between the *Ascaris* species indicated a majority of overlapping peak positions.

**Figure 9:**
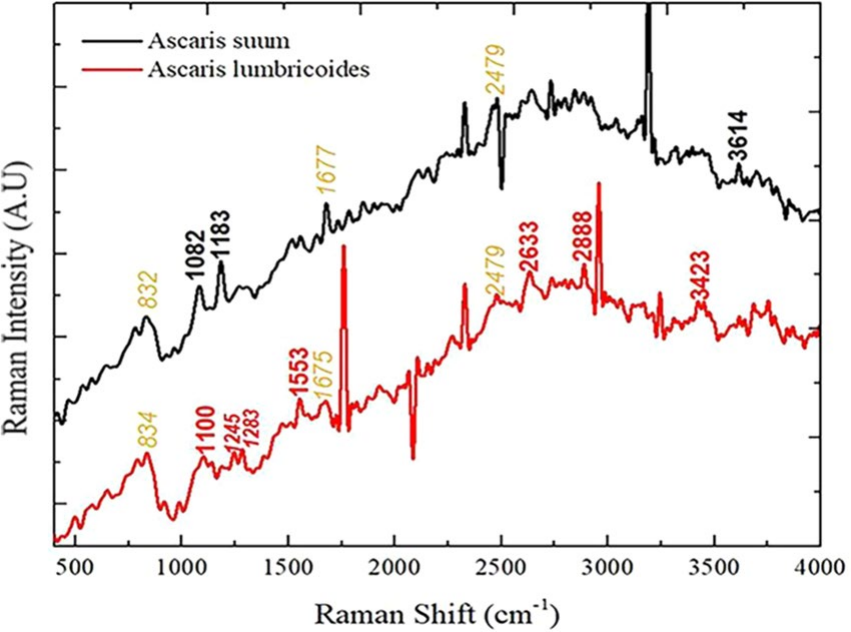
Raman spectra of *Ascaris lumbricoides* and *Ascaris suum* obtained using a Horiba Jobin Yvon at 532 nm laser, collected at 400–4000 cm^–1^ range. The spectra were obtained at 3 accumulations of 60 seconds at 40 mW laser power on each sample. The black highlighted text in the *Ascaris suum* spectrum indicates specific peaks which are characteristics to these eggs, while red highlighted text indicates specific peaks in the *Ascaris lumbricoides* eggs’ spectrum. The yellow italicized region represents similar peaks observed in species.

Similar peaks in both species are shown in yellow text in Fig. 9. The position of bands around 832 cm^−1^ and 835 cm^−1^ represents C-O-C stretching or C-C aliphatic chains and is associated with the presence of tyrosine of proteins^48^. The position for bands around 1677 cm^–^^1^ and 1675 cm^–^^1^ vibrations are associated with C = C vibrations and they represent amides, possibly proteins or chitins^48^. The peak at 2479 cm^−1^ indicates the region of O-H, N-H and C-H vibrations^48,49^.

Specific peaks to each species are shown in Supplementary Table 1. *Ascaris suum* have relatively prominent peaks at 1082 cm^−1^, 1183 cm^–1^ and around 3614 cm^−1^, indicating the presence of carbohydrates, proteins and lipids^49,50^. The eggs of *Ascaris lumbricoides* show peaks at 1100 cm^−1^, 1245 cm^−1^, 1283 cm^−1^, 1553 cm^−1^, 2633 cm^−1^, 2888 cm^−1^ and 3423 cm^−1^. These peaks indicate the presence of lipids and some percentage of proteins, carbohydrates and chitins^51^. *Ascaris lumbricoides* is reported to have more proportions of lipids (59%) and ascarosides (41%) compared to *Ascaris suum* where these are 36% and 7.9% respectively; this could account for the slight differences observed in Raman spectra (Fig. 9) and emission spectra (Fig. 7C)^51^. Due to the presence of higher proportion of lipids, eggs of *Ascaris lumbricoides* were more fluorescent as compared to *Ascaris suum* (Fig. 2).

**Figure 10:**
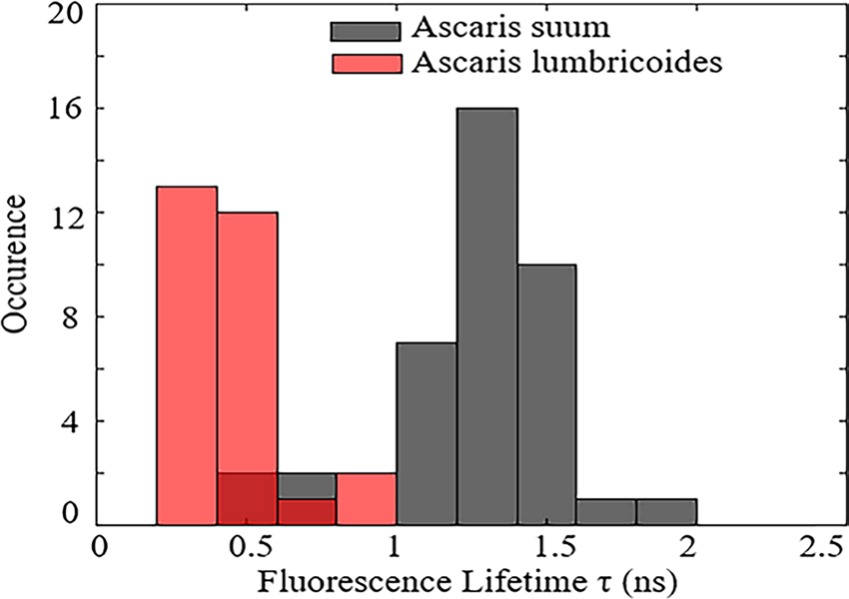
The lifetime of *Ascaris lumbricoides* and *Ascaris suum* obtained using a 532 nm pulsed laser with a 20 MHz repetition rate obtained at 25 µW laser power. The date set contained 28 to 39 different fluorescent sites from total of five to six eggs of each sample.

In the present study, we have performed lifetime measurements in order to differentiate species of nematodes i.e *Ascaris suum and Ascaris lumbricoides*, as shown in Fig. 10. The data set was taken from 39 fluorescent sites from a total of six different eggs in *Ascaris suum* while in *Ascaris lumbricoides*, data set was collected from 28 different sites from five different eggs. Promisingly, the lifetime of the two species were very different, with only 7% overlap for *Ascaris suum* to *Ascaris lumbricoides* and 10% overlap for *Ascaris lumbricoides* on *Ascaris suum*. The fluorescence lifetime value for *Ascaris suum* and *Ascaris lumbricoides* were 1.2 ± 0.3 ns and 0.4 ± 0.1 ns respectively. Lifetime is inversely proportion to emission intensity which accounts for the longer lifetime of *Ascaris suum* than *Ascaris lumbricoides*. Therefore, fluorescence spectra do not provide a high level of confidence for identification of *Ascaris suum* and *Ascaris lumbricoides* eggs; but when combined with lifetime measurements they can be classified with 90% confidence.

The lifetime measurements of species show that they can be distinguished from each other in real-time, thus offering a reliable method of identification. This methodology could assist the clinical method for diagnosis of *ascariasis*, in particular at the diagnostic stage (eggs) rather than infectious stage (juveniles). Compared with prior reports on detection of species^41,52^, the present study clearly distinguishes the species of nematodes with 90% certainty. To the best of our knowledge, there is no similar report on identification of species of nematodes through their lifetime measurements. The characterization reported here focuses on fluorescence spectroscopy analysis of different species of nematode eggs, with the objective of verifying that confocal microscopes can be effectively used to distinguish different genus and species of nematode eggs, by determining specific spectroscopic fingerprints.

#### Practical issues

##### Identification of species in sludge.

 So far, samples were prepared and purified in lab conditions. Next, we considered real non-purified samples of contaminated wastewater and soil that would occur at contaminated sites. Sewage sludge may contain many pathogens such as viruses, bacteria, protozoa and soil-transmitted helminths (STHs), and is disposed of as fertilizer for agricultural systems^53^. These pathogens are removed and killed by different chemical methods to avoid risk of human exposure^54^. However, *Ascaris* eggs are highly resistant to chemicals, heat and physical stress^55^. These *Ascaris* eggs are present in very high concentration in fertilizers and biosolids due to their ability to concentrate and accumulate together in sludge during wastewater treatment^2,56^. In the process of primary treatment, eggs of *Ascaris* settle more easily in sludge due to their larger size than bacteria and viruses, and it is estimated that on an average, 3000 *Ascaris* eggs/g are present in dry sludge^57^. Identification of *Ascaris* eggs from sludge is of major concern due to their heterogeneity^57^. In complex, heterogenous samples (soil particles and sludge) eggs can be easily trapped or attached to particles, making their identification extremely difficult.

We used confocal microscopy to identify *Ascaris* eggs in sludge samples using their intrinsic fluorescence properties. It was observed that eggs are even brighter than in clean samples. Supplementary Figure 5 shows the comparison of histograms of lifetimes obtained from stretched exponential fit for *Ascaris suum* and *Ascaris lumbricoides* eggs in a sludge sample. The overlap for lifetime for two species previously was 90% but in the presence of sludge it reduced to 80% (Supplementray Fig. 5) Therefore, lifetime measurement is a useful parameter to identify *Ascaris* eggs even in sludge samples with 80% confidence. These findings define principles for fabricating onsite devices with real-time responses that could identify these STHs in complex heterogenous samples.

##### Different populations of the eggs.

 In order to see whether the results are consistent with different population of eggs, lifetime and spectral analysis were performed on another population of *Ascaris lumbricoides* sample. It has been observed that lifetime is unchanged for different populations of *Ascaris lumbricoides*. Slight variation (red shift) in spectra has been observed. The same values for the lifetime and much the same spectra indicate that this combined emission properties parameters is a reliable identifier.

In this work, the intrinsic fluorescence properties of parasitic nematode eggs were investigated using a scanning confocal microscope. The identification and, in particular, the tracking of fluorescent features in bio-samples performed at room temperature, as presented in this paper, is advantageous. All eggs exhibited a bright green to red fluorescence in the UV and visible to near-infrared region with photostable counts for an extended period of time. Our results also showed that these parasites can be distinguished from each other by detecting specific optical signature in spectra of a single species. For distinguishing between species of nematodes, lifetime measurements provided the identification of parasites with 90% certainty in purified samples, and 80% confidence in environmental samples. Lifetime, as a single parameter, if confirmed to be unique for other parasitic eggs, would be the best candidate for future detection and identification devices. However, some species may have similar lifetime and in that case, the combination of spectral features and lifetime may be necessary. This is the first reported demonstration for identification of species of parasitic nematodes in environmental samples. The detection and identification of different species of nematodes require only few minutes and therefore this technique is highly applicable for real-time detection and identification. Once this technique automated it should even halve the time consumption taken for original measurements. These results are essential for identifying the species and will provide valuable information about public health risks associated with the eggs and aid in tracking the source of contamination. Our methodology offers sensitivity, specificity and non-invasiveness by utilizing the intrinsic fluorescence of these pathogens.

Therefore, the non-invasive optical method demonstrated in this work, that takes advantage of intrinsic fluorescence of nematodes, combined with the bright and photostable emission characteristics of eggs, may serve as the basis for development of devices for quick and reliable identificationof nematode eggs in water and soil, instead of relying on traditional methods that are laborious and time-consuming.

## Materials and Methods

### Egg origin and preparation

*Toxocara canis, Parascaris equorum* and *Oxyuris equi* eggs were provided by Dr. Otero from the department of parasitology within the Faculty of Veterinary Medicine in the National University of Mexico (UNAM). The technique used to identify the eggs by the National University of Mexico is light field optical microscopy looking for specific features in each of the eggs. Electron microscopy may also be used. In some cases, a culture is prepared, and the juvenile is developed.

These inactivated eggs were purified by grinding the female adult worms in saline solution and later kept in ethanol. *Ascaris lumbricoides* and *Ascaris suum* eggs were donated by Helen Williams from the School of Science, RMIT University. They were purified by the zinc sulphate concentration method^58^.

### Optical characterization

Samples of different species of nematode eggs were investigated by using a room temperature home-built scanning confocal microscopy which uses a Supercontinuum pulse laser (Fiannium, WhiteLase SC400). An excitation wavelength of λ = 532 nm was selected, and the laser beam was focused on the sample with a 100 × 0.9 NA objective lens. The fluorescence from the sample was filtered with a 532 nm laser notch filter before being detected. The egg samples on the cover glass substrates were mounted on a piezo controlled scanning stage facing the objective lens horizontally and assembled with *xyz* closed loop with a travel of 200 µm in each direction. Avalanche photodiodes (APD, SPCM-AQRH-14) were used to capture the fluorescence from the sample in the red and near infrared region. A spectrometer (Acton Spectra Pro 2300i with PIXIS100 camera) was employed for spectral analysis. For spectral acquisitions, 480 nm and 532 nm pulse lasers were used to excite the samples by using power range of 5 μW to 10 µW.

Time-resolved PL decay traces were obtained by correlation card (Picoquant, TimeHarp 260). The repetition rate for supercontinuum laser was set to 20 MHz with pulse width of 6 ps. Fluorescence decay traces were obtained by time-tagging the photon arrivals following the end of each pulse and averaging the signal over a total integration time of 30 s. The decay traces were then fitted to a stretched exponential decay model to extract the fluorescence lifetime. About 16 to 39 different points from five to eight eggs of same sample were measured for each species of egg.

It takes few minutes to collect the fluorescence and spectra from about 10 different spots. Then, it takes 15 minutes to measue the lifetime in around 20 to 30 different spots. Plotting spectra and fitting the lifetime to the stretched exponential fit takes another 15 minutes maximum. Therefore, the whole procedure takes around 40 minutes. However, this can be automated and in that case it would halve time-consumption.

Absorption spectral analysis: The absorbance spectra of the five different species of nematodes were measured using a Plate reader-SpectraMax Paradigm-Molecular device.

Spectral characterization at 80 K were done to see whether cryogenic measurements would affect identification of nematodes at species level. For this purpose, we used a Renishaw Invia Reflex micro-Raman spectrometer. The optical excitation was carried out using an Argon laser with a wavelength λ = 532 nm an acquisition time of 30 s. The measurements were performed using a 50× objective lens (0.75 NA) and a stage mounted cryostat assisting in fluorescence spectra acquisition at 80 K temperature (i.e. a liquid-nitrogen temperature). The use of CCD array coupled with a single grating of 1800-lines mm^-1^ is allowed for a fast acquisition of fluorescence spectral maps over a broad spectral range (520–1000 nm).

Raman spectroscopy was carried out on *Ascaris lumbricoides* and *Ascaris suum* eggs. Samples were suspended in sterile water and around ~30 µl of the deactivated sample was dropped on a glass slide and covered with a cover slip. A Horiba Jobin Yvon Raman Spectroscope was used with an excitation laser of 532 nm. A CCD detector and X50 LWD objective (0.5 NA) was selected to view the samples. Light was dispersed at a grating of 1800gr/mm and spectra were collected at a range of 400–4000 cm^–1^. The laser was focused at the surface of the egg for three accumulations of 60 seconds at 40 mW power. Spectra were collected from two different spots from three different eggs of each species.

For 3*D* imaging, *Ascaris lumbricoides* sample was mounted in glycerol on a slide and covered with coverslip. For visualization, LSM 880 Airyscan laser scanning microscope (Carl Zeiss Microscopy) equipped with PlanApo 20×/0.8 objective lens was used. The laser and filter were 561 nm/ BP 495–550 + LP 570 nm and pinhole was set at 384 µm. For analysis Zeiss LSM 880 using ZEN black software was applied. The collective lambda mode picture (composed of superimposed 21 and 14 pictures) was obtained using wavelength of 405 nm (Fig. 4) and 561 nm (Fig. 5) respectively. The brightness and contrast were further adjusted using ImageJ.

Wide Field Imaging. The wide-field analysis was undertaken on a commercial fluorescence microscope (Olympus Ix83) adapted for wide-field imaging, connected to ultra-sensitive Hamamtsu Flash 4.0 Camera and DP275MP camera. The samples were observed under the white light as well as illuminated with 390 nm UV laser and 560 nm laser. The samples were magnified with 40x objective lens.

## Supplementary information

Supplementary information.
