# Supplementary material for: Real-time detection and identification of nematode eggs genus and species through optical imaging
Source: Sci Rep. 2020 Apr 29;10:7219. doi: 10.1038/s41598-020-63747-5 (PMC7190725; doi:10.1038/s41598-020-63747-5)
Supplement: Supplementary file 1 — Supplementary information. [file 41598_2020_63747_MOESM1_ESM.docx]

**Supplementary information**

**Real time detection and Identification of nematode eggs genus and species through optical imaging**

Farah Qazi^1^, Asma Khalid ^1,2^, Arpita Poddar^3^, Jean-Philippe Tetienne^1^, Athavan Nadarajah^1^, Arturo Aburto-Medina^4^, Esmaeil Shahsavari^4^, Ravi Shukla^3^, Steven Prawer^1^, Andrew S Ball^4^ and Snjezana Tomljenovic-Hanic^1^

1. School of Physics, University of Melbourne, Parkville, 3010, Australia

2. College of Science, Engineering and Health, RMIT University, Melbourne, Victoria 3001, Australia

3. Ian Potter NanoBiosensing Facility, NanoBiotechnology Research Laboratory (NBRL), School of Science, RMIT University, Melbourne, Victoria 3001, Australia

4. Centre for Environmental Sustainability and Remediation (EnSuRe), School of Science, RMIT University, Bundoora, Victoria 3083, Australia

**S1: Wide-field Microscopy**

For optical imaging of nematode eggs and juveniles, wide-field microscopy was performed.


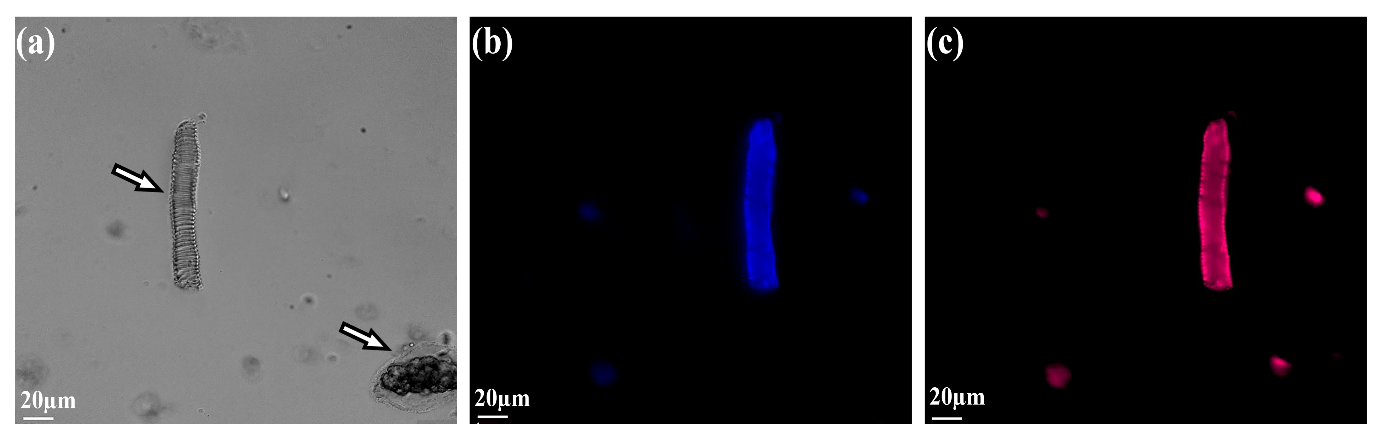


Figure S1: The wide-field microscopy image of *Ascaris lumbricoides* juvenile under (a) white light (b) UV 390 nm light (c) 560 nm light. The arrows are pointing towards juvenile and egg of *Ascaris lumbricoides*


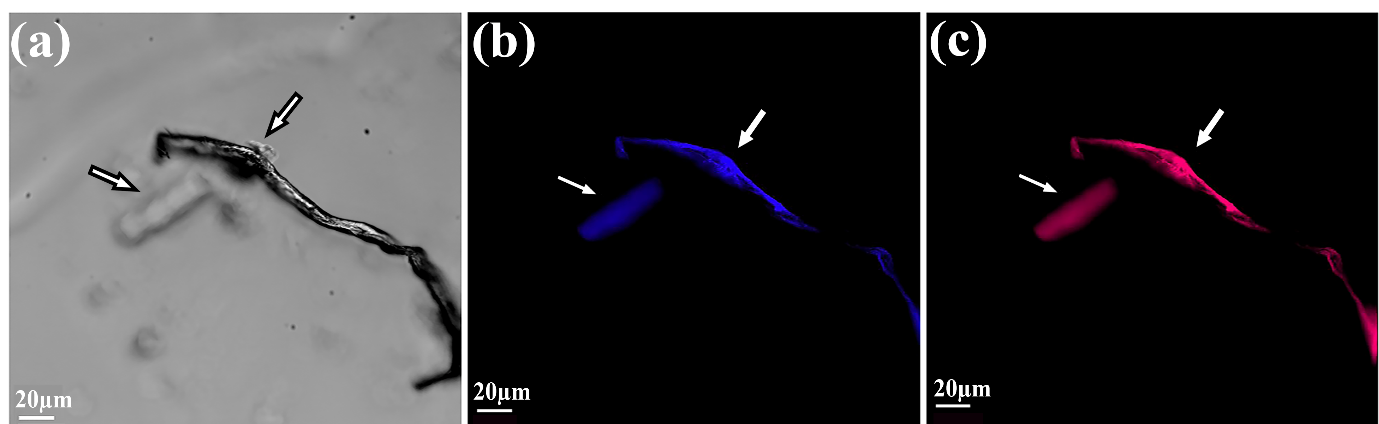


Figure S2: The wide-field microscopy image of *Ascaris suum* juveniles under (a) white light (b) UV 390 nm light (c) 560 nm light. The arrows are pointing towards juveniles.


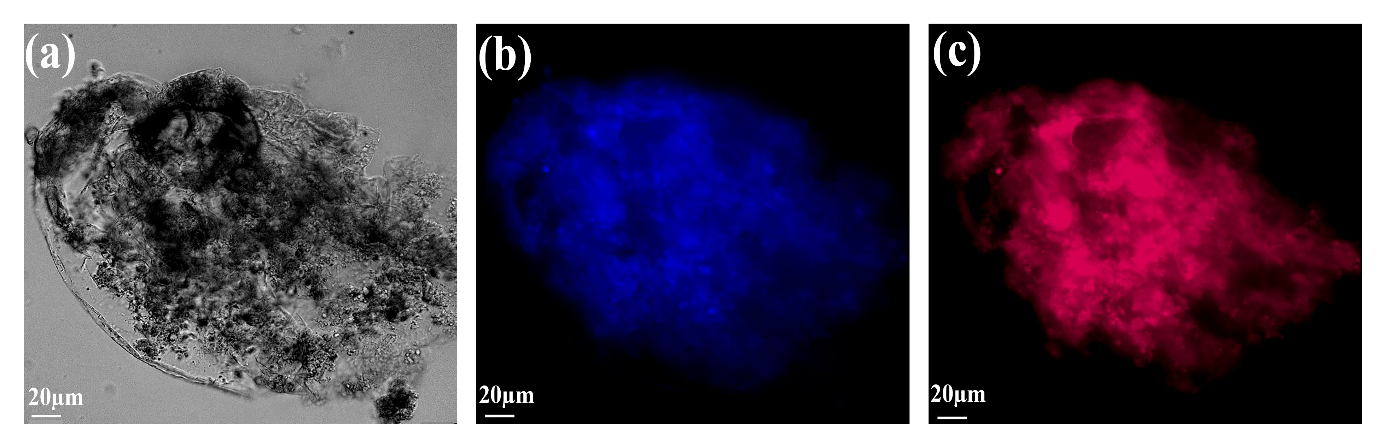


Figure S3: The wide-field microscopy image of *Ascaris suum* egg under (a) white light (b) UV 390 nm light (c) 560 nm light


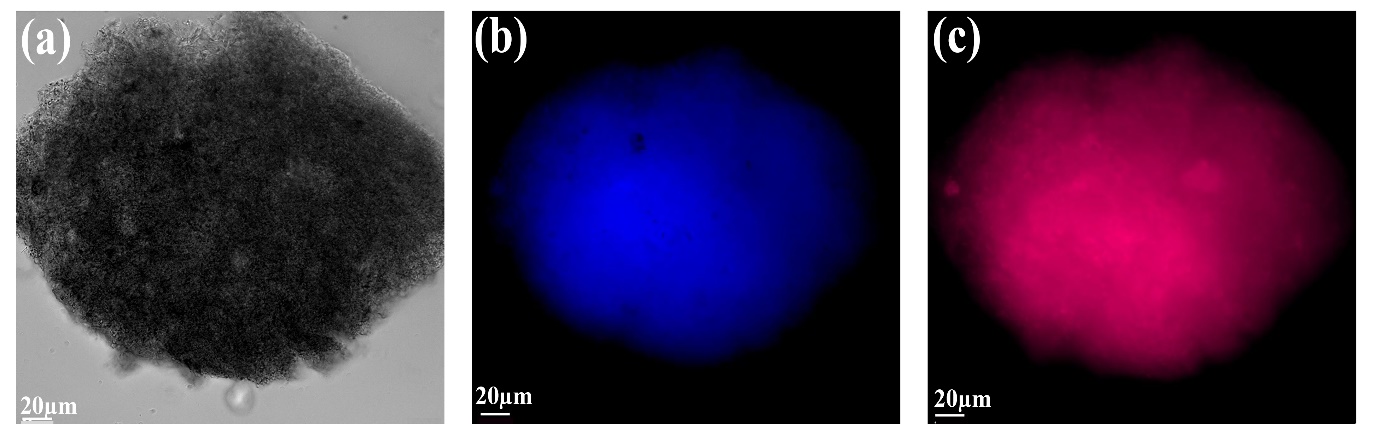


Figure S4: The wide-field microscopy image of *Toxocara canis* egg under (a) white light (b) UV 390 nm light (c) 560 nm light

**S2: Raman Measurements**

The Raman measurements were performed to see the chemical composition of species. The table shows the tentative biochemical composition difference observed in species.

| Species | Raman shifts (cm^-1^) | Peak assignment | Tentative Composition | Tentative Biochemical composition |
| --- | --- | --- | --- | --- |
| *Ascaris suum* | 1082 | strong aromatic stretching (Phe) | Carbohydrates,  possible ascarosides | 14-24.9 %, (ascarosides 7.9%) ^1^ |
|  | 1183 | ν C–C aliphatic chains and ν C-O | Polysaccharides, triglycerides, | 24.8 % ^1^ |
|  | 3614 | O-H, N-H | Possibly cellulose, amide |  |
| *Ascaris lumbricoides* | 1100 | Aromatic, Alicyclic, ν C–C aliphatic chains | Polysaccharides, triglycerides | Lipids 59% ^2^ |
|  | 1245 | ν C–C aliphatic chains, ν C-O | Polysaccharides |  |
|  | 1283 | ν (C-C) Alicyclic, Aliphatic chains, unsaturated chains of fatty acids | Unsaturated triglycerides |  |
|  | 1553 | ν(N=N) Aliphatics chains, ν C=C | Amide II, Proteins | Proteins 25% ^3^ |
|  | 2633 | ν S-H, ν C-H | Proteins |  |
|  | 2888 | ν C-H stretching | Chitins and Lipids, ascarosides |  |
|  | 3423 | ν N-H, ν O-H | Cellulose |  |

Table 1: Vibrational bands specific to the species obtained from Raman spectra of *Ascaris suum* and *Ascaris lumbricoides* indicating biochemical composition based on peak positions.

⁕ ν represents stretching

**S3: Lifetime measurements in sludge**

We used confocal microscopy on *Ascaris* samples present in sludge as shown in figure S2.


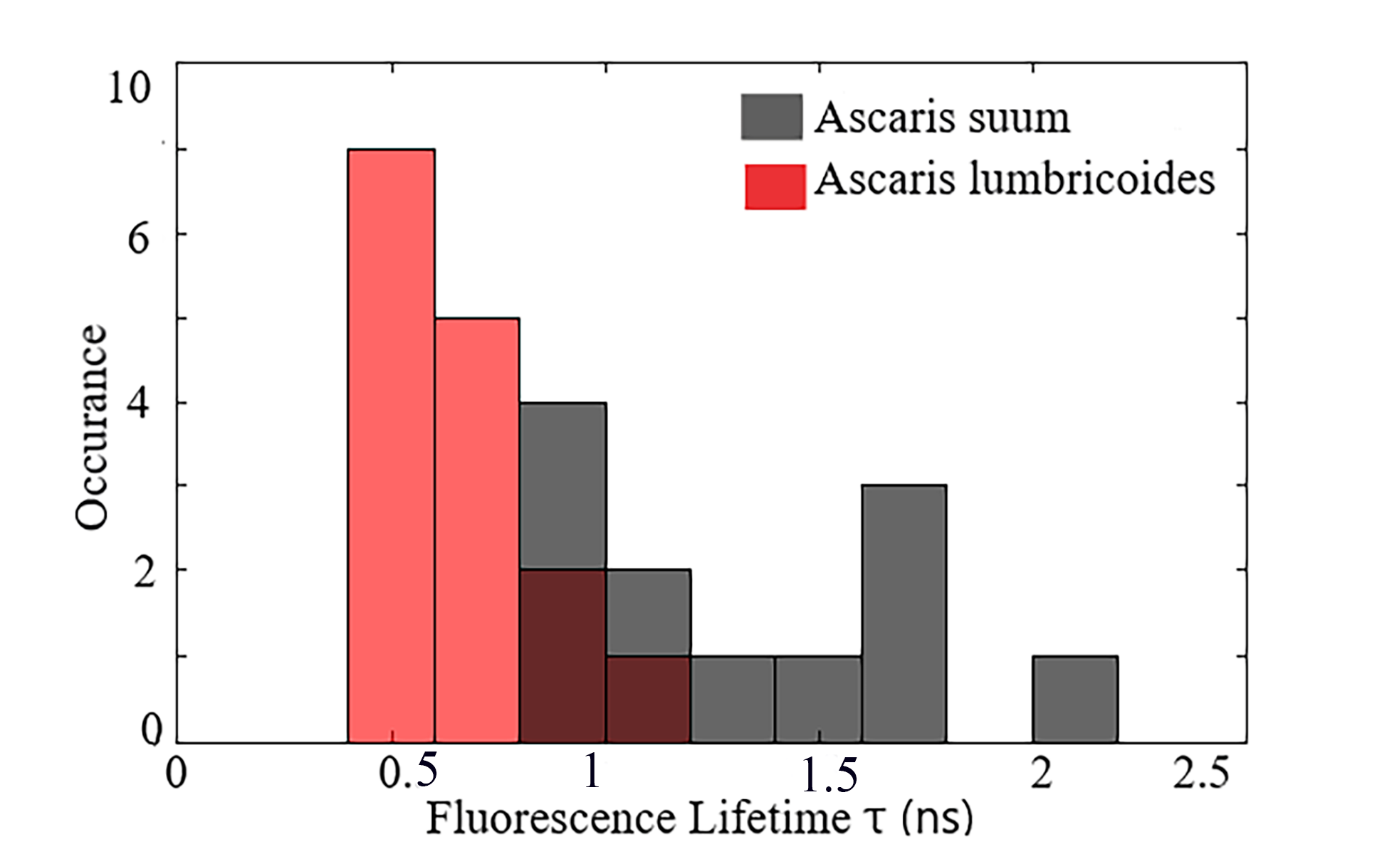


Figure S5: The lifetime of *Ascaris lumbricoides* and *Ascaris suum* in a sludge sample obtained using a 532 nm pulsed laser with a 20 MHz repetition rate obtained at 5µW.

Each data set contained 15 to 15 fluorescent sites from a total of two different eggs from each sample. Note that the five times lower power was used in comparison to purified sample in section ‘identification of species’ because photoluminescence intensity is strongly affected of the samples and influenced by their environment ^4^. In previous case, eggs were surrounded by air with the refractive index of n=1, whereas sludge has much larger refractive index of n=1.33 to 1.5. Those optical differences dictate the condition under measurements are taken. The mean fluorescence lifetime values of *Ascaris suum* and *Ascaris lumbricoides* were approximately 1.3±0.4 ns and 0.6±0.1 ns (mean ± standard deviation), respectively.

**References**

1 Quilès, F., Balandier, J.-Y., Capizzi-Banas, S. J. A. & chemistry, b. In situ characterisation of a microorganism surface by Raman microspectroscopy: the shell of Ascaris eggs. *Anal. Bioanal. Chem.* **386**, 249-255 (2006).

2 Fairbairn, D. & Passey, B. I. The lipid components in the vitelline membrane of Ascaris lumbricoides eggs. *Can. J. Biochem. Physiol.* **33**, 130-134 (1955).

3 Fairbairn, D. J. E. p. *The biochemistry of Ascaris*. **6**, 491-554 (1957).

4 Chung, K. & Tomljenovic-Hanic, S. J. N. Emission properties of fluorescent nanoparticles determined by their optical environment. *Nanomaterials* **5**, 895-905 (2015).
